# Factors Affecting Fertility Rate in Iran (Panel Data 1966-2013): A Survey Study 

**Published:** 2017-09

**Authors:** Asma Sabermahani, Reza Goudarzi, Sarah Nasiri

**Affiliations:** 1 Health Services Management Research Center, Institute for Futures Studies in Health, Kerman University of Medical Sciences, Kerman, Iran; 2 Modeling in Health Research Center, Institute for Futures Studies in Health, Kerman University of Medical Sciences, Kerman, Iran; 3 Research center for Social Determinants of Health, Jahrom University of Medical Sciences, Jahrom, Iran

**Keywords:** Fertility Rate, Panel, Population Policies, Economic Policies

## Abstract

**Objective:** Population and its corresponding problems are among multidimensional and complicated issues of human communities and their related features are the basis for making any plan or policy. Fertility, as one of the principle components of population growth, is an issue that has always been taken into consideration and extensive research has been carried out to recognize factors affecting on it. Therefore, the authors decided to study the most important factors influencing fertility rate in Iran by conducting a longitudinal study and considering the effect of various time periods on its population changes.

**Materials and methods:** This is a descriptive-analytic study. Its required information is a combination of cross-sectional and time series data (panel data) that were extracted from 1966 to 2013 from Iran’s population categorized by the country’s 24 provinces and from statistical yearbooks of Statistical Center of Iran and Organization of Civil Registration. The final estimations were made using Eviews 7 and STATA 12 software. Findings showed that variables of marriage, women’s level of education, unemployment, population policies, Sunni population, economic policies and annual expenses of households have influenced the fertility rate.

**Results:** Based on the research results, marriage and women’s level of education respectively had the most positive and the most negative effects on the fertility rate. Then, unemployment, family planning policies, policies of paying cash subsidies and total annual household expenses had reverse effects on the fertility rate and the policies of paying cash subsidies and Sunni population had positive effects on the fertility rate.

**Conclusion:** In order to make policies of increasing fertility rate effective by governmental and politicians’ planning, more attention should be paid to providing conditions for marriage and reducing unemployment.

## Introduction

Population and its corresponding problems are among multidimensional and complicated issues of human communities and their related features are the basis for making any plan or policy. In fact, population is one of the important economic and social components in any community and population growth (fertility status) should be under control in order to reach sustainable development (1).

The phenomenon of fertility is one of the important natural events of population and one of the important elements of population growth, for the increase of which, some countries apply incentive policies and some others apply punitive ones. Certainty and stability in population issues do not have many applications and no specific reason can be used to determine their dimensions. On the other hand, there is not a fixed and permanent relation between their variables (2).

Recognition of fertility dynamics and factors influencing it, which form the most important population component in today’s world, are inevitable necessities in development programs. This is particularly of high importance for developing countries that are more concerned about development and growth. As a developing country, Iran has faced a rapid reduction in fertility during the last decade (3).

Although the common attitude is that reduction of fertility in recent years is a result of economic pressures and after overcoming the economic problems, fertility can be expected to increase again but factors such as urbanization, marriage age, Improve in education especially in women, public family planning policies and world culture will definitely lead to a more reduction of fertility in the upcoming decade and it is expected that if families enjoy a better economic condition, they will care more about the quality of their children and will not think about the number of them (4).

Based on the census done in 2006, the amount of fertility throughout the country was 1.8 children per mother and country’s population growth was 1.62 and in the census done in 2011, the average of country’s population growth was announced to be 1.29 (5). While the critical fertility rate (total amount of fertility that prevents from reduction and elimination of population over time) is about 2.4 children. It means that the level of fertility is lower than the level of survival or critical level. In other words, country’s population will fall into the path of decline after the aforementioned year (6).

Fertility, as one of the major components of population growth is an issue that has always been the center of attention and extensive research has been carried out in the field of recognizing the factors influencing it in Iran and in foreign countries (7). Examples include: Heydari-Sooreshjani et al. (8), Moosayi et al. (9), Shiri and Bidarian (10), Ziayi-Bigdeli et al. (2), Kalantari et al. (11), Rahnavard et al. (12), Adsera and Menendez (13), Hondroyiannis (14), Engelhardtet and Prskawetz (15) and Kreyenfeld (16) have carried out studies in this regard.

The present study is different from other studies of the same type in some aspects which include the use of Panel approach and the study of the issue in a macro view by the use of general information about provinces, not the families separately, as well as the use of questionnaires. Unlike being abundant in studies carried out in other countries, this type of research has had no precedence in Iran to the best knowledge of researchers. According to the aforementioned issues, the most important factors influencing the rate of fertility in Iran were decided to be investigated by a study that could be carried out over time and by considering different periods of population changes.

## Materials and methods

The present paper is a socioeconomic study based on a descriptive-analytical model. The required information to carry out the study was a combination of cross-sectional and time series data (panel data) that were extracted from 1966 to 2013 from all residents of country differentiated by 24 provinces and from statistical yearbooks of Statistical Center of Iran and the country’s Civil Registration Organization. As the number of Iran provinces was changed during periods, researchers had to use the least number at the beginning of the period and integrated data for divided provinces in other years. Panel data is one of the approaches of econometrics and is a combination of cross-sectional and time series data. The limitations that exist in each one of the time series models (autocorrelation) and cross-sectional data (heteroskedasticity) can be reduced in a panel model. By combining these two groups in a panel, and with the increase of the number of observations and the degree of freedom, the alignment problem between explanatory variables will decrease and the efficiency of econometric estimation will increase (17).

In the present study, fertility rate (FR) as a dependent variable [Total fertility rate = Σ (five-yearage-specific birth rates for females aged 15 to 49)] has been analyzed against four groups of descriptive variables. The first group, economic indexes of provinces include variables that describe the economic status of the provinces namely unemployment rate (UnempR) and the family’s total annual expenses (FaTExp). The second group constitutes the economic policies of the government which include cash and non-cash subsidy policies (Sub). It’s expected that paying subsidy lead to more fertility because of Motivational effect in economic aspect. The third group includes the population policies of the government which are the family planning policies (FaPlan). The fourth group, socio-economic indexes of provinces, including marriage rate (MarR), women’s education rate (EduWomR) and the ratio of Sunni population to the whole population in the province (SunR), It is thought, Sunni population has cultural and religious specificities that lead to more fertility so more ratio of this group in provinces population may have direct relation with fertility rate . It should be mentioned that in specification process many other variables were studied and were deleted from the model base on goodness of fit test. For example women employment, men education, Finally the applied multiple linear regression model was as follows:

*FR*_it_=*α*_i_+*β*_1_*MarR*_it_+*β*_2_*EduWomR*_it_+*β*_3_*UnempR*_it_+*β*_4_*FaPlan*_it_+*β*_5_*SunR*_it_+*β*_6_*Sub*_it_+*β*_7_*FaTExp*_it_+*ε*_it_

In this regression, “i” stands for provinces and “t” shows the years. Also β coefficient of each variable indicates its level of effect related to that dependent variable and α coefficient is the intercept of the model.

*α*_i_ is an intercept which is random. In linear regression, distribution of error term is considered normal. As in “basic econometrics” book written by Damodar Gujarati it’s mentioned, this consideration has many benefits. “u is frequently assumed to follow a normal distribution, there is no theoretical reason for the selection of this or other distributional forms for u” (18) (19).

Programs and policies of paying cash and non-cash subsidy and its absence have been divided into six periods in dummy variable forms. D_1_: not paying the subsidy, D_2_: paying non-cash subsidy (ten coupons), D_3_: paying non-cash subsidy (five coupons), D_4_: paying non-cash subsidy (four coupons), D_5_: paying non-cash subsidy (three coupons) and D_6_: paying cash subsidy.

Family planning policies were analyzed during two periods in the form of dummy variables. D_1_: presence of family planning policies and D_2_: absence of public family planning policies for population reduction. 

Sunni population has also been divided into three categories of dummy variables: D_1_: provinces with less than five percent of Sunni population, D_2_: provinces with Sunni population of five to fifty percent and D_3_: provinces with more than fifty percent of Sunni population.

Finally, by the use of related tests, the appropriate estimation method (from among three methods of least-squares integration, fixed effects and random effects) was selected. First Chow Test (H_0_ = Pooled, H_1_ = Fix Effects) and Breusch-Pagan Test (H_0_ = Pooled, H_1_ = Random Effects) were used to determine the type of model. Hausman Test (H_0 _= Random Effects, H_1 _= Fix Effects) was used to decide between fixed effects and random effects models and when the type of the model was determined, the final estimation was done by Eviews 7 and STATA 12.

Six models were estimated to show factors affecting on fertility rate. In the first one unemployment, marriage rate, family planning policy, women’s education rate and family’s total annual expenses were surveyed. In the second one, family’s food and nonfood annual expenses were surveyed instead of family’s total annual expenses. Urban and rural nonfood annual expenses were surveyed in the third model. In two next models subsidy policies were examined and in the last model, the effect of sunni population were researched.

## Results

Set of data in time periods of 1966 to 2013 was collected from 24 provinces and formed 1152 observations. Then different models were studied based on the variables extracted from the texts. Based on the results, the best models were selected in terms of compatibility with the theories of significance and goodness to fit and were reported as follows.

Table 1 shows the results of Chow Test, Breusch Pagan and Hausman Tests for economic indexes, economic and population policies as well as socio-cultural indexes.

**Table 1:** Tests results to choose appropriate estimation method

**Tests**	**Parameters**	**Amount**	**Degree of freedom**	**Significance**
Chow Test (f test)	F	21.76	(23, 1123)	0.0001
Breusch Pagan Test	Chi2	2135.91	(01)	0.0001
Hausman Test	Chi2	5.73	(4)	0.2206

Chow and Breusch-Pagan tests were applied separately for all purposes and the necessity of using panel data method against least-squares integration was examined for the estimation. As the amounts in table 1 show, when the zero hypothesis is rejected, panel data method (fixed and random effects) is confirmed. In the next step, Hausman Test was applied to select one method between the two panel data estimation methods of fixed and random effects methods and it was used separately for all purposes. Based on Hausman Test results that are presented in table 1, the calculated probability indicates that zero hypothesis is accepted and random effects model is confirmed for estimation.

Three models were estimated to study the effect of economic indexes on fertility rate and two models were presented to study the effect of economic policies on the fertility rate. In order to study the effect of population policies of the government on fertility rate, the variable data of family planning policies were constantly added in all models. Ultimately, the data related to marriage variable in all models, educated women’s variable in most models and Sunni population in one model were estimated to study the effects of the socio-economic indexes on fertility rate. They are presented in table 2.

The obtained results of all models show that the coefficient of the effect of marriage on fertility rate has been positive and it has had the most direct effect on fertility rate. The effect of the coefficient obtained by the ratio of educated women population to the province population was negative in all models and this coefficient has had the highest reverse effect among all other coefficients on fertility rate. The effect coefficient of unemployment rate has been negative in all models and it is indicative of the reverse effect of unemployment rate on fertility rate. Results of population policies models indicate that the effect coefficient of family planning policies has been negative and has had a reverse effect on fertility rate (the forth variable in the table 2).

Based on the obtained results from the model, provinces whose Sunni population is between five to fifty percent and provinces with Sunni population of more than fifty percent had higher rate of fertility, compared to the provinces with Sunni population of less than five percent.

According to the results of economic policies models, effect coefficients of the first three periods during which the policy of paying non-cash subsidy (coupons for ten, five and four items) was applied and the number of coupons were high was positive which means they had a direct effect on fertility rate. However, the last two periods during which the policy of paying non-cash subsidy had reached its lowest level (coupons for three items) and the policy of paying cash subsidy was applied, had negative effect coefficients and a reverse effect on fertility rate.

Based on the results of the model of economic indexes, effect coefficient of family’s total annual expenses on fertility rate was lower than the coefficient of all other variables. In order to ensure the obtained results, the index of family’s total annual expenses was divided into annual food and non-food expenses of a family and the effect of non-food expenses of a family that was a representative of expenses such as rental of a house, transportation and peripheral expenses such as taking care of children were studied. Results showed that these expenses also have negative but little effect on fertility rate over time. In the next step, family’s annual non-food expenses were divided into urban and rural aspects. After estimating the model, the coefficient of this model turned out to be negative for both urban and rural households that showed the reverse but small effect of expenses on fertility rate.

## Discussion

In this study, attempts were made to make use of appropriate models to evaluate the collected data. However, the present study has some limitations. There might be other variables that can affect fertility rate and have not been considered in this study due to the absence of some other data, instances of which may be variables like life expectancy, health indexes, singleness and the ratio of ethnic minorities. In addition, inaccessibility to the data that were influential in fertility rate and inaccessibility to the required data regarding the households were some of the limitations in the present study. On the other hand, because data resources did not overlap or were not consistent with each other, the obtained results might have been influenced. Finally, the present research estimated and analyzed different models, which is unprecedented.

Based on the obtained results, marriage has been the main and most important factor influencing fertility rate. Other studies confirm the results of this research like the study conducted by Iman et al. (20) which showed that there is a positive relation and correlation between teenage girls’ marriage rate and their childbearing in urban areas. 

**Table 2:** Results of the models about factors affecting fertility rate in Iran

**Model**		**Model 1**		**Model 2**		**Model 3**		**Model 4**		**Model 5**		**Model 6**	
**Variable**		**Coefficient**	**S.E**	**Coefficient**	**S.E**	**Coefficient**	**S.E**	**Coefficient**	**S.E**	**Coefficient**	**S.E**	**Coefficient**	**S.E**
UnempR		-0.059	0.016	-0.057	0.016	-0.056	0.016	-0.020*	0.015			-0.012*	0.025
MarR		5.789	0.091	5.788	0.091	5.789	0.090	6.088	0.086	6.088	0.086	4.304	0.126
EduWomR		-0.194	0.005	-0.193	0.005	-0.193	0.005	-0.215	0.007	-0.215	0.007		
FaPlan		-0.010	0.000	-0.010	0.001	-0.010	0.000	-0.005	0.001	-0.005	0.001	-0.026	0.001
FaTExp		-3.38×10^-11^	4.63×10^-12^					-1.05×10^-11^	5.54×10^-12^	-1.10×10^-11^	5.53×10^-12^	-1.26×10^-10^	6.00×10^-12^
FaFExp				2.83×10^-12^^*^	3.56×10^-11^								
FaNFExp				-5.22×10^-11^	1.83×10^-11^								
UFaNFExp						-1.07×10^-11^^*^	3.31×10^-11^						
RFaNFExp						-1.23×10^-10^	5.83×10^-11^						
Sub	D1							0.009	0.001	0.009	0.001		
	D2							0.011	0.001	0.012	0.001		
	D3							0.002*	0.001	0.002*	0.001		
	D4							-0.002*	0.001	-0.002*	0.001		
	D5							-0.003*	0.002	-0.003*	0.002		
SunR	D1											0.003*	0.002
	D2											0.005*	0.003
Cons		0.041	0.001	0.040	0.001	0.040	0.001	0.033	0.001	0.032	0.001	0.025	0.002
Goodness of Fit Criteria	R2	0.76		0.76		0.76		0.78		0.78		0.52	
	AIC	-6379		-6379		-6385		-6480		-6474		-5578	

* Insignificant coefficients

Findings of studies of Hondroyiannis (14) and Jafari et al. (21) indicated that an increase in the number of marriages will lead to the higher rate of fertility.

With the improvement of women’s level of education and their entering the society, marriage age has increased and their inclination to have children has decreased and on the other hand, information and accessibility to tools for fertility control and pregnancy prevention has increased. Also better job opportunities will make women feel independent economically, socially and mentally. Educated women emphasize the quality of children more than their quantity. Therefore, they will face less fertility. According to numerous studies such as Rahnavard et al. (12), Akaberi et al. (22), Noroozi (23), Moti-Haghshenas (24), Adibi-Sadeh et al. (7) and Odwe et al. (25), strong and significant effect of women’s level of education was recognized as the most important factor and the main accelerator of fertility reduction.

Unemployment rate is one of the most important economic causes of fertility reduction. Results of other studies confirmed this fact. Seemingly, it was shown in the studies of Kreyenfeld (16), Adsera (26), Adsera and Menendez (13) and Jafari et al. (21) that unemployment significantly delays fertility.

During the years when family planning policies were implemented, these policies were effective and fertility rate has reduced. Research findings of Kalantari et al. (11) (27) indicate the fact that spreading family planning services in the country, positive attitude and women’s agreement to use birth control methods have reduced fertility.

Various religious minorities have different attitudes about fertility. Having more Sunni population has had positive effects on fertility rate. Findings of studies done by Mahmoodian and Nobakht (28), Hashemian and Mohamadi-Gol (29) have shown higher levels of fertility among Sunni women compared to Shia women and its significance is another confirmation to the current research.

At the time when cash subsidy policy was implemented, unlike the theory that paying cash subsidy must encourage people to higher levels of fertility, coefficient of the period of policy of paying cash subsidy had become negative due to other reasons such as an increase in unemployment resulted from paying cash subsidy, ineffective allocation of them or factors that have influenced the level of fertility over time. Interpreting these results, one can say that the power of negative effect of variables such as unemployment and women’s education rate in the society has been so high that has superseded the positive effect of paying cash subsidy on fertility rate during the period. Mention to educated women attitudes about fertility importance and preparing good condition and supportive environment for parents can be so helpful. Also, paying cash subsidy has temporarily reduced the financial load of more children and has not been considered a helpful fundamental policy in the long run. Research results of Kalwij (30) also indicate that the increase of subsidy for children doesn’t have a significant effect on fertility. This policy is an allowance for direct expenses of children, not an allowance for expenses of childbearing opportunity that has become more important for fertility decisions in the recent decades.

Unlike the public’s belief that one of the most important factors of reducing fertility rate is increasing expenses and income status of households, one can see that the negative index of family’s total annual expenses has not had any remarkable effect on the reduction of fertility. Other studies are also consistent with the results of this research about negative sign of this variable. For example, Heydari-Sooreshjani et al. (8) and Hezar-Jaribi and Abaspoor (31) have stated that increasing life expenses and economic pressure will lead to a reduction in fertility. The study of Kodzi et al. (32) also showed that the probability of fertility will decrease in bad economic situation of a family. In the study of Dettling and Kearney (33), it was clarified that household expenses will lead to negative effect of expenses that will reduce birth rate.

## Conclusion

In order to make policies of increasing fertility rate effective by governmental and politicians’ planning, more attention should be paid to providing conditions for marriage and reducing unemployment. Getting married and starting a family can be possible in a society if unemployment is reduced through creating jobs, especially for men, providing accommodations, increasing public awareness and scientific justification of marriage and fertility logic because as long as the youth are unemployed and have no income, that is until they live in an insecure economic condition, they cannot think about marriage and delay it. Consequently, childbearing will be postponed, too.
